# An Optimized Approach to Multistage Permanent Deformation Testing of Granular Materials

**DOI:** 10.3390/ma17143384

**Published:** 2024-07-09

**Authors:** Erdrick Pérez-González, Jean-Pascal Bilodeau

**Affiliations:** Department of Civil and Water Engineering, Université Laval, Québec, QC G1V 0A6, Canada

**Keywords:** deformation, performance, granular, laboratory, pavements

## Abstract

Accurately characterizing permanent deformation in granular materials subjected to cyclic loading is crucial for pavement design. This paper introduces an alternative approach to characterizing permanent deformation in a framework that reduces the number of load cycle repetitions by applying an alternative analytical strategy based on plastic strain rate variation over time. The methodology uses a cycle-hardening approach to establish correlations between short-term (post-compaction) and long-term (shakedown state) plastic strain accumulation. This alternative approach provides an efficient means to accelerate the characterization of permanent deformation, ensuring the integrity and validity of the assessment in a more time-efficient and resource-optimized way.

## 1. Introduction

Describing the permanent deformation behavior of unbound granular material (UGM) is essential for predicting pavement performance [1,2,3]. UGMs form the base and subbase layers in flexible pavements, and an inadequate response to the stresses induced by repeated traffic loading can result in excessive accumulation of permanent deformation and rutting. Understanding and effectively managing this phenomenon is needed for the efficient and sustainable design of pavements and other geotechnical infrastructure subjected to a high number of repeated load cycles.

Various test equipment and methods are available for the performance characterization of UGM [4,5]. One of the most used is the triaxial testing device, widely recognized in the civil engineering community. This equipment can be classified into several categories according to its purpose. For example, the true triaxial test has been noted in the literature for considering the mean principle stress, although its operational complexity is significantly increased [6,7].

On the other hand, the triaxial compression test has been a long-standing tool in evaluating soils and unbound materials [8]. This test provides information on fundamental material properties, outperforming other geotechnical methods. Among these tests, the repeated load triaxial test (RLTT) has gained popularity in pavement engineering due to its ability to accurately simulate repeated dynamic loading of vehicles [9]. More recently, the precision unbound material analyzer (PUMA) has emerged as an option for performing dynamic repetitive load testing [10,11]. This equipment can measure axial and radial strain and deformation of UGM under repetitive loading. Although it offers the advantage of providing a dynamic confining pressure to characterize the actual pressure under wheel loading, its use is still not widespread.

In practice, the RLTT is the most commonly used laboratory method to determine the permanent deformation properties of UGM [12,13]. During this test, the response of a UGM specimen under repeated loading is divided into resilient (recoverable) deformation and permanent (unrecoverable) deformation. The recoverable behavior allows the characterization of the resilient modulus, and the accumulation of permanent deformation can be characterized by different constitutive models, which should be able to reliably predict the deformation behavior of the materials considering the various factors influencing it [14,15].

The RLTT involves the controlled application of axial and confining loads on a specimen to simulate the dynamic damage process expected in the field. Throughout the test, the axial deformations of the granular material are recorded to describe the progressive accumulation of permanent deformation over time, typically measured by the number of load applications. A standard practice in performing this type of test is using a single specimen to evaluate various stress conditions, known as a multistage test [12]. One of the challenges that can be found in using the multistage condition is to accurately assess the influence of stress history on permanent deformation accumulation, which, if not done properly, can compromise the overall representativeness of the analyses performed [3,16].

One tool available to analyze results from a multistage RLTT is the strain-hardening approach, in which an analytical correction to the permanent deformation measured in the laboratory is performed. For this, an equivalent number of load cycles ($N_{i}^{eq}$) needs to be determined for each stress stage. An example of the formulation of this approach is represented by Equation (1) [17]:

(1) $$\varepsilon_{p}=\alpha {\left(N_{i}^{0}+N_{i}^{eq}\right)}^{\beta }$$

where $\varepsilon_{p}$ is the accumulated permanent deformation, α and β are model parameters, $N_{i}^{0}$ represents the number of cycles in the stress path i from zero, and $N_{i}^{eq}$ is an adjustment factor for the stress path i. The formulation outlined in Equation (1) follows a permanent deformation prediction model expressed as $\varepsilon_{p}=\alpha N^{\beta }$. In this model, the parameters of the strain-hardening approach substitute the variable N to correct the influence of the stress history in permanent deformation development. This approach is adaptable to any predictive model incorporating the number of cycles (N) in its formulation [17].

Multistage RLTT test protocols require a high number of load repetitions per stress state to ensure the robustness of the trend described by the permanent deformation development in the UGM; however, this same condition makes it difficult to describe the material performance accurately without considering the effect of stress history. Even applying the strain-hardening approach, assessing the validity of the $N_{i}^{eq}$ value can pose challenges, particularly in the later stages of the test, where the material may experience stresses lower than those generating a significant permanent stain rate due to advanced deformation progress in the sample, leading to the value of $N_{i}^{eq}$ tending to infinite.

The UGM performance is sensitive to the direction of the imposed stress path [1,18]; therefore, it is reasonable to assume that the same material could exhibit different performances when subjected to different stress paths, even under the same stress state. This will depend on the material’s stress history and the number of loading applications to which it has been subjected.

This paper introduces an optimized approach to evaluate permanent deformation in UGM under multistage cyclic loading conditions. The proposed optimization improves the conditions to be evaluated in the laboratory to allow a better interpretation of the permanent deformation behavior under a high number of load repetitions. This optimization is based on using the plastic strain rate as a reference parameter to improve the understanding of the evolution of the permanent deformation, facilitating a more efficient evaluation of the response of granular materials.

## 2. Background

**Figure 1:**
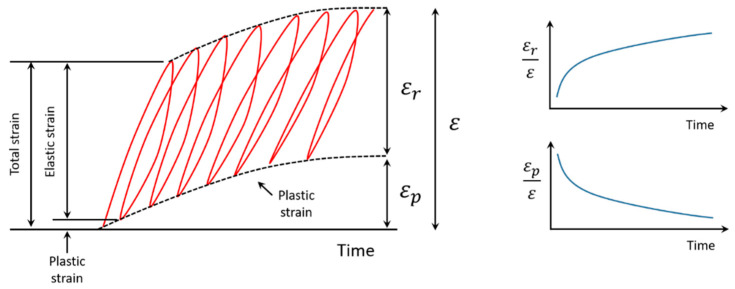
Resilient and plastic strain behavior in a material subjected to cyclic loading.

Deformation in UGM subjected to repetitive loading comprises two distinct elements: the elastic component, characterized by resilient strain ($\varepsilon_{r}$), and the plastic component, defined by plastic strain ($\varepsilon_{p}$), as illustrated in Figure 1. In this behavior, the proportion of permanent deformation ($\varepsilon_{p}/\varepsilon$) tends to exceed that associated with resilient deformation ($\varepsilon_{r}/\varepsilon$) in the initial loading cycles. However, as the number of applied loads increases, the deformations finally reach elastic equilibrium ($\varepsilon \approx \varepsilon_{r}$).

The description of the permanent deformation (PD) performance of UGM is usually addressed through laboratory characterization, using two main analysis approaches: (i) the use of benchmark indicators to assess the acceptability of material performance and (ii) the use of empirical prediction models, which are calibrated with laboratory results, to estimate the accumulation of PD under specific stress states and environmental conditions. Research on permanent deformation has been the subject of extensive study, including characterization using approaches such as the shakedown theory [16,19,20,21,22,23].

Shakedown theory states that materials subjected to cyclic loading will exhibit a performance determined by the stress state, eventually reaching a state known as “shakedown” after a significant number of load repetitions [20,21]. The hypothesis adopted by the shakedown theory was found to be applicable to the behavior of UGM. The main utility of considering this theory as a framework for analysis of UGM is its relatively simple ability to establish indicators of material acceptability for the stress states to which it is expected to be subjected. Shakedown theory categorizes PD into the following distinct ranges:

- Range A, characterized by a predominantly elastic response, occurs at sufficiently low-stress levels.
- Range B is observed when loads moderately exceed the elastic limit, resulting in a direct relationship between cumulative PD and the number of repeated loads.
- Range C is marked by a significant increase in cumulative PD, leading to shear deformation and potential collapse.

Range A and Range B are generally acceptable for flexible pavements, while Range C should be avoided. Identifying the boundaries between different shakedown ranges mainly involves two approaches described in the literature. One is an empirical approach based on the plastic strain rate, where the boundaries between shakedown ranges are determined from reference values obtained by laboratory observations [1,21,22,24,25]. The second approach considers that the limits of shakedown states can be calculated by defining the critical point of failure [26,27]. According to this approach, a granular material subjected to cyclic vehicular loading has inherent shakedown limits that can be defined by parameters analogous to the cohesion (c) and friction angle (ϕ) used in the Mohr–Coulomb model [28,29].

European standards [12] have adopted limits proposed by Werkmeister et al. [19], relying on PD values between 3000 and 5000 cycles. The boundaries between shakedown ranges are defined based on the average deformation rate in these cycles, as shown in Table 1.

The European specification uses an equation to model the stress defining the boundary between shakedown ranges [12]. This is rational since having a model that can predict PD enables the analysis of the shakedown state of the material under specific conditions.

**Figure 2:**
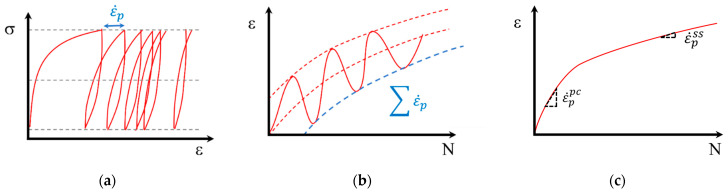
Illustrative representation of PD: (**a**) implicit approach, (**b**) explicit approach, (**c**) characteristic plastic strain rates in UGMs.

Numerous models have been developed to predict permanent deformation and characterize the elastoplastic behavior of granular materials under repetitive loading [2,30,31,32]. These models revolve around two fundamental approaches: implicit and explicit. Implicit methods involve calculating plastic strains for each loading cycle the material undergoes, represented as the plastic strain rate (${\dot{\varepsilon }}_{p}=\partial \varepsilon_{p}/\partial N$). In contrast, explicit calculation methods determine the deformation path for each loading step or cycle by calculating the accumulated plastic strain ($\varepsilon_{p}$) in pseudo-time, with the number of cycles (N) replacing time. In practice, explicit models are the most widely used, mainly because implicit methods accumulate errors with each load cycle, which limits their reliable application to cases with a small number of load applications (i.e., N less than 50) [33]. Both implicit and explicit approaches are empirical, employing mathematical formulations to describe laboratory observations, as illustrated in Figure 2a,b.

**Figure 3:**
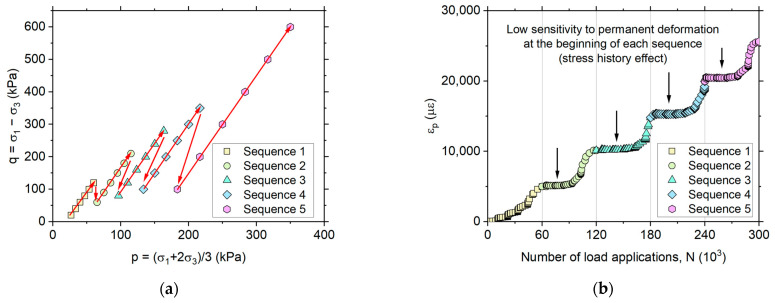
Multistage permanent deformation test: (**a**) stress path (low-stress level) in the European specification [12], (**b**) typical results in a granitic unbound granular base.

Both implicit and explicit prediction models require calibration from laboratory results. For this purpose, various stress state conditions are used, generally defined by a deviatoric stress ($q=\sigma_{1}-\sigma_{3}$) and a mean stress ($p=\left[\sigma_{1}+2\sigma_{3}\right]/3$), where $\sigma_{1}$ and $\sigma_{3}$ are the major and minor principal stresses, respectively. The stress path for a multistage RLTT is specified in the European specification [12], and some typical test results are illustrated in Figure 3. 

Considering a material subjected to repetitive loading with relatively constant stress states, two different deformation phases can be distinguished according to the strain rate: (i) the post-compaction phase, represented by ${\dot{\varepsilon }}_{p}^{pc}$, where a high plastic strain rate is observed due to a rapid adjustment in the granular material structure in the initial stages of PD accumulation, and (ii) the equilibrium phase behavior, described by ${\dot{\varepsilon }}_{p}^{ss}$, characterized by a relatively constant plastic strain rate after the application of a large number of load repetitions. This last phase can be identified as the shakedown equilibrium state. An illustrative representation of these concepts is shown in Figure 2c.

Figure 3a shows the five loading sequences specified in EN 13286-7 [12]. Each stage involves different stress state conditions, defined by the combination of q and p, with 10^4^ loading cycles performed per stress state for a total of 6 × 10^4^ loading cycles per sequence, and 30 × 10^4^ load cycles for a complete test. The main difference between sequences lies in the increase of the confining stress ($\sigma_{3}$). In multistage RLTT, the sequential load application implies that the material in some cases may experience lower stress states than previously applied. In Figure 3b, an apparent reduction in the material’s susceptibility to permanent deformation in the initial loading cycles is observed when moving from one sequence to another (i.e., sequences 2, 3, 4 and 5), compared to the first sequence. This observation underscores the impact of stress history on material behavior.

Research has confirmed that the plastic strain rate between cycles 100 and 1000 in an RLTT adequately represents the behaviour during the post-compaction phase of granular materials [33]. Similarly, it has been established that the average plastic strain rate measured in the laboratory between cycles 3000 and 5000 represents the shakedown phase of the material [12]. Although the ranges to adequately represent the different deformation phases, i.e., post-compaction and shakedown state, were defined empirically, a review of laboratory results along with full-scale field measurements suggests that the strain rate between 3000 and 5000 loading cycles does not necessarily reflect the equilibrium strain rate of the material at a high number of loading repetitions [22]. This finding implies that relying only on a single range of load applications (i.e., 3000 to 5000) to characterize long-term behavior may be insufficient. However, this has been an accepted practice, mainly due to the limitations of laboratory testing capabilities, where subjecting a specimen to millions of load repetitions is often infeasible [19,22,28,29].

A simple correlation between plastic strain rates in the post-compaction (${\dot{\varepsilon }}_{p}^{pc}$) and the shakedown state (${\dot{\varepsilon }}_{p}^{ss}$) has been previously described by Pérez-González et al. [3].

(2) $${\dot{\varepsilon }}_{p}^{ss}=0.175\times {\dot{\varepsilon }}_{p}^{pc}$$

This correlation has been established from multiple RLTTs on various UGMs in Quebec, Canada. In this case, ${\dot{\varepsilon }}_{p}^{pc}$ is associated with the average plastic strain rate between 100 and 1000 load cycles, while ${\dot{\varepsilon }}_{p}^{ss}$ is associated with the plastic strain rate between 3000 and 5000 load cycles. More recently, a correlation between ${\dot{\varepsilon }}_{p}^{pc}$ and ${\dot{\varepsilon }}_{p}^{ss}$ has been established by probabilistic analysis from the results of full-scale tests performed with a heavy vehicle simulator [16].

Experimental evidence of a correlation between plastic strain rates within the post-compaction and shakedown phases, as shown in Equation (2), indicates that with repeated loading, the behavior of the material normalizes to a specific plastic strain rate and that this strain rate can be inferred from the response of the material to initial load applications. Understanding this property of UGM lays the foundation for an optimized approach to PD characterization in multistage RLTT. Focusing on early plastic strain rate analysis can reduce the impact of specimen stress history on test results more appropriately. The objective of this study is to use the post-compaction plastic strain characteristic of the UGMs as a reference to establish an analysis protocol to predict the long-term behavior of the permanent deformation, resulting in an optimization of the test protocol followed in the laboratory.

## 3. Materials and Methods

**Figure 4:**
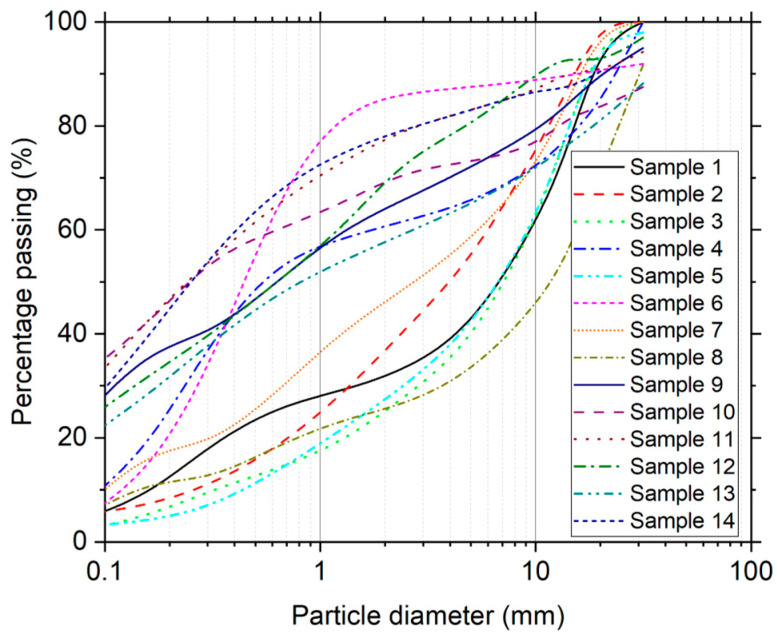
Grain size distribution of UGMs.

In this study, the behavior of various UGMs was evaluated in the laboratory with the objective of describing normalized trends between the ${\dot{\varepsilon }}_{p}^{pc}$ and the plastic deformation rates measured at a high number of load repetitions. Fourteen sand and gravel materials were considered in the analysis, whose descriptive characteristics are shown in Table 2. Based on these results, an optimized laboratory protocol can be proposed for multistage RLTT. The particle size of the UGMs was analyzed following ASTM C136/C136M [34] (see Figure 4). Maximum dry density (MDD) and optimum water content (OWC) were determined according to ASTM D1557 [35]. 

Multistage RLTT was performed using the low-stress level procedure of the European standard EN13286-7 [12]. A sinusoidal impulse with a loading frequency of 1 Hz was applied without a rest period. The strain-hardening approach presented by Rahman and Erlingsson [31] was used to improve the interpretation of the laboratory results. A power model, as expressed in Equation (1), was considered for the analysis. The quadratic error minimization technique was applied to identify the material parameters α, β and $N_{i}^{eq}$ for each stress state tested, which were subsequently incorporated into the proposed analysis. Only models with an R^2^ fit greater than 0.95 were retained for analysis. A total of 37 stress state results were discarded by this process. A result with a lower than desired R^2^ can be attributed to the limited ability of the power model to adequately fit the behavior in a condition highly affected by the stress history, where permanent deformation, even in the first loading cycles, is already associated with a shakedown state, making it difficult to determine the $N_{i}^{eq}$ parameter. Although more complex models could be used to mitigate this problem, in this study it is considered that, despite the discarded proportion (11.5% of the total data), the described trends are still representative of typical conditions of granular materials.

This paper aims to study the variation of the plastic strain rate in materials at a high accumulation of load repetitions. Using the parameters α and β determined for each test conditions, the characteristic plastic strain rates for a defined number of load repetitions (${{\dot{\varepsilon }}_{p}}^{N}$) can be calculated using Equation (3):

(3) $${{\dot{\varepsilon }}_{p}}^{N}=\frac{\alpha }{N_{i}-N_{i-1}}\left[{\left(N_{i}\right)}^{\beta }-{\left(N_{i-1}\right)}^{\beta }\right]$$

For the RLTT, the specimens were compacted into six individual sub-layers of approximately 50 mm using a vibratory hammer. The sample dimensions were 152.4 mm for the diameter and 300 mm for the height. The compaction water content was fixed for each sample as the OWC. A rubber membrane was used around the samples to confine the top and bottom of the sample, and to ensure sealing vacuum grease and four O-rings were used. The drain valve on the top and bottom of the specimens was kept open during the tests (drained conditions). To measure axial displacements, the samples were instrumented with two LVDTs on the sample, placed in the middle 200 mm of the compacted samples.

## 4. Results

This section provides an overview of the experimental results and their interpretation, which reveal two key behaviors relevant to optimizing the RLTT laboratory protocol under multistage conditions: (i) a cyclic hardening and (ii) the change in plastic behavior based on the stress history. These aspects will be developed in the following sections.

### 4.1. Cyclic-Hardening 

The hardening of granular materials is a phenomenon that occurs when materials undergo plastic deformation due to loading [37]. It means that the materials become stronger and more resistant to further deformation. By addressing the hardening of the UGMs as a function of variations in the plastic strain rate, a proportional reduction in this indicator is expected as the number of load cycles applied to the material increases. A correlation can be established to explain the hardening phenomenon in granular materials using a generalized formulation of Equation (2), as presented in Equation (4):

(4) $${\dot{\varepsilon }}_{p}^{N}=\eta \times {\dot{\varepsilon }}_{p}^{pc}$$

where ${\dot{\varepsilon }}_{p}^{N}$ represents the plastic strain rate after N cycles of load application, and η is a hardening function yet to be defined. This mathematical formulation represents a directly proportional relationship between ${\dot{\varepsilon }}_{p}^{pc}$, a constant for the material under given stress conditions, and the material stiffness, represented by its susceptibility to permanent deformation during its lifetime.

**Figure 5:**
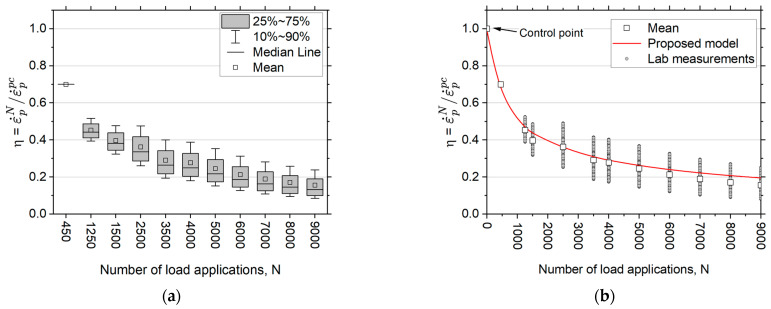
Change in material stiffness, represented by η. (**a**) Experimental results, (**b**) cycling hardening model (Equation (5)).

The evolution of η as a function of the accumulation of load repetitions can be analyzed by the laboratory results, covering 252 different combinations of materials and stress states. Figure 5a illustrates η as a function of the number of load repetitions (N), where η represents the ratio of ${\dot{\varepsilon }}_{p}^{N}$ to ${\dot{\varepsilon }}_{p}^{pc}$ (see Equation (4)). The plastic strain rate has been calculated within the interval ±450 of the N value reported in the figure, and the values of η are associated with probable ranges of occurrence according to experimental observations.

The experimental results reveal a consistent trend in the variation of η in the materials studied, indicating a behavioral basis for formulating a description of cycle hardening in the UGMs. One control point was incorporated into the experimental data to build the model to mitigate extreme predictions in the initial cycle numbers (see Figure 5b). Using the mean values of η, Equation (5) was derived:

(5) $$\eta =\frac{1}{1+{\left(\frac{N}{1000}\right)}^{0.645}}$$

The cyclic hardening model presented in Equation (5) shows that as the number of loading cycles, represented by N, approaches infinity, the value of η converges towards 0. This suggests that a granular material, subjected to a certain level of stress, will exhibit predominantly elastic behavior after a high number of load repetitions, which is in accordance with what has been described in the literature on the subject.

### 4.2. Plastic Behavior with Stress History

The study of permanent deformation in UGMs intimately depends on the stress state and the stress history. In multistage RLTT, addressing the effect of stress history poses a significant challenge in interpreting results [3]. To allow the use of a correlation between the long-term performance of a UGM and its behavior in the initial state (${\dot{\varepsilon }}_{p}^{pc}$), it is necessary that the sequence of loads evaluated in the laboratory allows the quantification of ${\dot{\varepsilon }}_{p}^{pc}$ reliably, limiting the effect of past stress states. To consider this, a virtual yield surface has been adopted in this study to facilitate the interpretation of the stress history effect in the laboratory protocol.

A yield surface is a concept used to characterize the response of materials to stress. Generally, it represents the boundary between elastic and plastic behavior, and its shape can evolve during plastic deformation [37]. Stress states within the yield surface indicate that the granular material behaves elastically. However, upon reaching the surface boundary, the material undergoes plastic deformation.

Based on the concept of yield surface, a reference surface is developed in this study to analytically separate the stress states in which granular materials will exhibit predominantly post-compaction behavior from those with shakedown behavior. It is important to note that this surface is not intended to indicate material failure, but rather a change in the development of permanent deformation after a high number of loading repetitions. This surface will hereafter be identified as the “threshold surface”, associated with the threshold between post-compaction and shakedown behaviour with respect to plastic strain accumulation.

**Figure 6:**
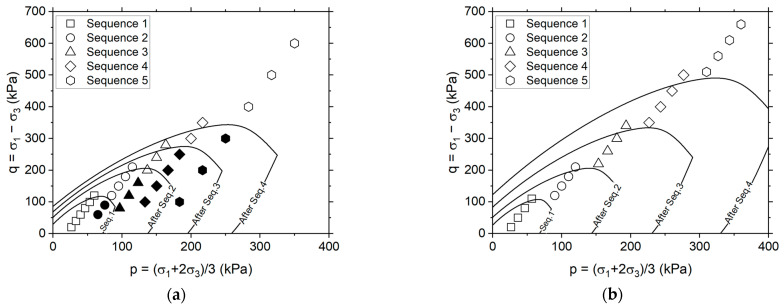
Evolution of the threshold surface after loading sequences: (**a**) European standard, low stress level [12]; (**b**) proposed stress path.

An elliptical threshold surface was developed from experimental observations to study the stress history phenomena. Within this surface, the stress states allow the determination of ${\dot{\varepsilon }}_{p}^{pc}$ within the first 1000 loading cycles. The Cartesian coordinates of the threshold surface are described by Equations (6) and (7):

(6) $$q_{i}=q\sin\left(\alpha_{i}+\frac{\pi }{4}\right)$$

(7) $$p_{i}=p\left(\frac{1}{2}+\cos\alpha_{i}\right)$$

where, q and p represent the deviatoric and mean stresses associated with the stress state, respectively, while α is a term ranging from 0 to 2π. Figure 6 shows the evolution of the threshold surface after different loading sequences considered in this study.

Figure 6a shows the rationale behind the threshold surface formulation based on the laboratory results typically observed in the UGM when applying the European standard. In this representation, the stress states highlighted in black are those assumed to be influenced by the stress history. This is derived from the analysis of typical experimental results in UGM; in these stress states, the plastic strain rates have been found to converge to stability during the first few cycles of repeated loading. This influence results in a reduction of the plastic strain accumulation, its behavior approaching more closely the ${\dot{\varepsilon }}_{p}^{ss}$ condition. 

The depiction shown in Figure 6a is designed to ensure that the stress states highlighted in black fall systematically within the threshold surface. This indicates that behaviour in this domain is expected to be different from that observed in a post-compaction state. Taking advantage of this feature, a stress path can be defined so that the stress states remain outside this hypothetical surface. This will ensure that laboratory testing facilitates a clear evaluation of ${\dot{\varepsilon }}_{p}^{pc}$, thus allowing the use of Equation (4) in the analysis of UGM performance. The stress paths proposed in this paper are presented in Figure 6b and detailed in Table 3.

The stress states outlined for each stress path, as specified in Table 3, ensure that the stress states consistently maintain incremental conditions while preserving conditions similar to those defined by the European specification. This approach also ensures alignment with currently accepted practices. For example, it maintains an increment of $\sigma_{3}$ between sequences of 30 kPa and increments of q between 30 kPa and 50 kPa within each sequence. 

## 5. Optimized Multi-State RLTT Protocol

Based on the above experimental results, a multistage RLTT protocol can be introduced, considering new stress paths while employing a reduced number of loading repetitions. Using the loading sequences specified in Table 3, each with 1000 loading cycles, is considered optimal. This optimization reduces 30 stress states requiring 10,000 load repetitions to 20 stress states requiring 1000 load repetitions. Consequently, the typical test duration of 83 h can be shortened to a rapid test completed in less than 6 h. This optimization not only maximizes laboratory efficiency but also facilitates faster analysis, thereby improving the ability to evaluate other components of the UGMs, such as variability within the same stress conditions, in a comparable time frame (e.g., duplicate specimens).

The optimized multistage RLTT protocol allows effective characterization of the ${\dot{\varepsilon }}_{p}^{pc}$ in the UGM. This indicator serves as a basis for analyzing two fundamental aspects in the study of the UGM: (i) the resistance of the material to deformation induced by repeated loading, which correlates with the states described in the shakedown theory, and (ii) the accumulated permanent deformation. The first aspect concerns the overall stability of the material under repeated loading, while the second focuses on predicting the extent of plastic strain accumulation after a given number of load repetitions. The analysis approaches proposed in the optimized multistage RLTT protocol framework are described in the following sections.

### 5.1. Stability of UGM under Repeated Loads

As previously described, the shakedown theory, which characterizes the deformation behavior in three ranges (i.e., A, B and C), is an accepted reference for the characterization of UGM under repeated loading [20,21,28,29]. By accurately determining the shakedown state, engineers can refine pavement designs to meet performance standards while minimizing construction and maintenance costs.

The proposed laboratory protocol, referred to as rapid multistage RLTT hereafter, focuses on the determination of ${\dot{\varepsilon }}_{p}^{pc}$ as the primary indicator. This parameter can be used to identify the shakedown state of the UGM. Equation (4) facilitates the calculation of ${\dot{\varepsilon }}_{p}^{pc}$, which can be associated with the shakedown states observed in practice (see Table 1). To achieve this correlation, a value of N = 4000 load cycles represent the range that characterizes the shakedown state in the current specifications (i.e., between 3000 and 5000 load cycles). Table 4 shows the reference values suggested under the proposed protocol.

Equations (6) and (7) can be used to define a boundary surface delimiting the transition between these states in a q−p space. In addition, along with the above, the accumulation of permanent deformation over a large number of load repetitions can be calculated using Equation (4), which involves the partial sum of each loading cycle, as represented in Equation (8):

(8) $$\varepsilon_{p}=\sum_{i=1}^{N}\left(\frac{1}{1+{\left(\frac{N}{1000}\right)}^{0.645}}\right)\times {\dot{\varepsilon }}_{p}^{pc}$$

**Figure 7:**
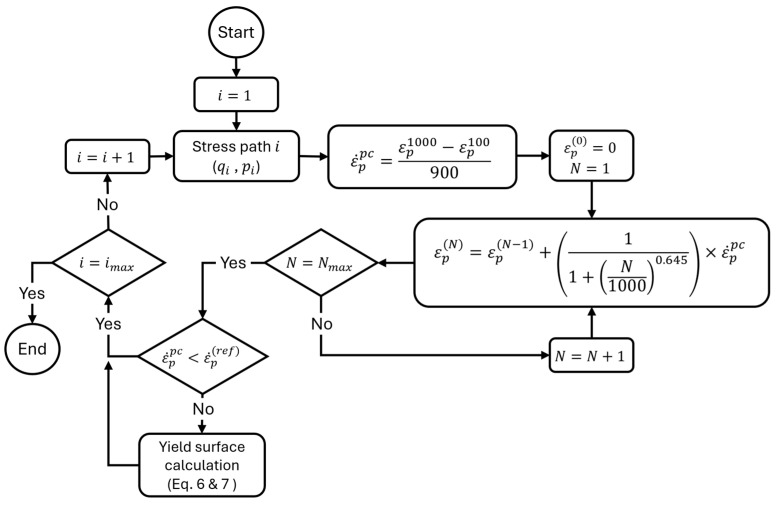
Flowchart for the analysis of UGM from multistage RLTT.

Figure 7 presents the flowchart to be followed to apply this methodology effectively. This protocol adaptation allows sufficient information to be obtained to characterize long-term permanent deformation behavior effectively. This approach implies substantial time savings and offers an alternative tool to evaluate the behavior of granular materials, significantly improving the efficiency of laboratory testing under multistage conditions.

### 5.2. Validation

**Figure 8:**
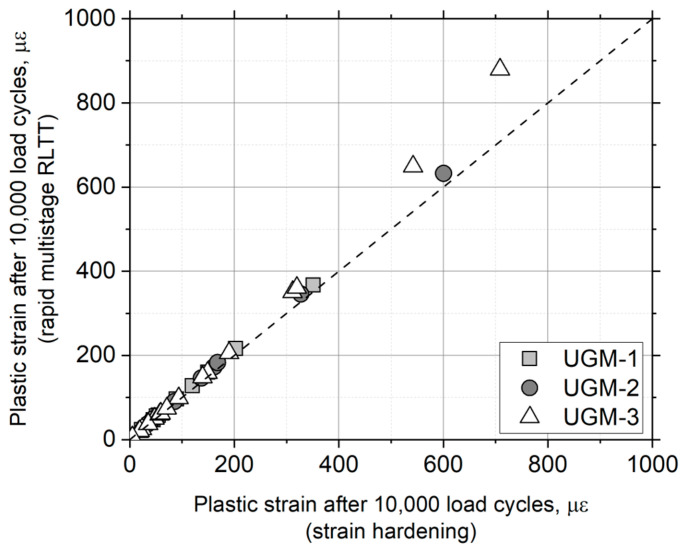
Comparison between laboratory measurements using conventional protocol and estimation performed using fast multistage RLTT protocol.

To validate the proposed approach, laboratory results of three different materials, which were subjected to the standard test protocol, were analyzed. From the laboratory measurements, the analysis using the strain-hardening approach and the analysis process described in Figure 7 were applied. The properties of the UGMs, as well as the identified shakedown state, are shown in Table 5. Also, Figure 8 compares the predictions of permanent strain accumulation after 10^4^ load repetitions.

As shown in Table 5, the three granular materials considered for validation, named UGM-1, UGM-2 and UGM-3, were subjected to a limited number of stress states under an incremental stress condition, applying nine stress paths to UGM-1 and UGM-2, and 14 to UGM-3, with higher stress levels in the latter case. UGM-3 was exposed to higher stress states to explore a shakedown state other than the “A” state. This condition also allows one to identify if the increase of stresses in the material results in significant variations in the predictions made by the proposed method. The criteria described in Table 1 and the proposal in Table 4 were equivalent to identify the shakedown state of the material.

Figure 8 shows a good consistency between the strain-hardening approach and the method proposed in this paper. The consistency between predictions and laboratory measurements is clear, with a coefficient of fit (R^2^) of 0.973. This analysis demonstrates that the proposed approach is robust and accurately represents the behavior of granular materials in laboratory tests under multistage conditions.

## 6. Discussion

The proposal of a method to reduce the number of loading cycles and stress paths in multistage RLTT, based on the plastic strain rate measured between 100 and 1000 cycles, represents an interesting optimization to characterize permanent deformation in UGM. This approach is based on the observation that the plastic strain rate during the first loading repetitions can provide valuable information on the susceptibility of the material to permanent deformation, as highlighted in previous studies [23,33].

The practical application of this method has several key implications. First, it allows a significant reduction in the time and resources required to evaluate the behavior of materials in different stress states. This efficiency is particularly relevant in situations where laboratory resources are limited, or rapid evaluation of multiple loading conditions is required.

In the validation of the proposed approach, it is observed that for predictions after 10^4^ load repetitions and relatively high plastic strains (greater than 400 με), the method tends to overestimate the values measured in the laboratory and analyzed with the strain-hardening approach. This behavior is more noticeable in the case of UGM-3 (see Figure 8). This discrepancy can be attributed to the lower amount of data available at this strain level at the time of model development, which causes the model to estimate a higher susceptibility of the material compared to the laboratory results. This behavior is specifically associated with stress paths under high stress conditions, where the variability in the data is greater and the model tends to extrapolate, resulting in an overestimation of the plastic deformation.

The general concept of using a global hardening model, as described in Equation (5), while useful for practical purposes, has the associated limitation that the performance predicted using this approach will be based on an average of the performance of the materials. However, even considering this limitation, gains in laboratory efficiency can be achieved, so that the rapid multistage RLTT, as described in this article, can be used as an initial assessment to indicate whether further analysis of the material is needed.

Although the results are promising, it is essential to recognize the limitations of the study. External validation and exploration of a wider diversity of materials and stress conditions are crucial steps to confirm the general applicability of the proposed method.

## 7. Conclusions

The RLTT is a valuable tool for evaluating the performance of granular materials. However, these tests involve a significant investment of time and resources, which can be challenging when exploring a wide range of conditions such as compaction, saturation and stress levels.

This paper proposes a method to optimize multistage RLTT. It consists of quantifying the plastic strain rate during initial loading cycles, which provides an early indicator of material behavior. Subsequently, a hardening model is employed to predict the long-term behavior of the UGM. Using reference limits for the plastic strain rate, the stability of the material can be identified using shakedown theory.

This approach achieves a 90% reduction in the time required to characterize the behavior of granular materials. Validation with laboratory results demonstrates strong consistency between predictions and actual measurements, supporting the effectiveness and accuracy of the proposed method. Ultimately, this methodology offers an efficient optimization of laboratory testing, allowing for a faster evaluation of the behavior of materials under multi-stage conditions.

The approach of using ${\dot{\varepsilon }}_{p}^{pc}$ to predict long-term permanent deformation in UGMs has been validated using a limited number of stress states. A stress path proposal has been formulated to reduce the number of load repetitions to 1000 per stress state, but its validation requires a more detailed study that exceeds the limits defined for this article.

Despite the identified limitations, such as the use of simplified model to determine a general trend of material hardening, and the overestimation of plastic strains greater than 400 microstrains, the proposed approach remains valuable, providing a robust tool for predicting the behavior of granular materials under various loading conditions. The identification of these limitations also provides an opportunity to improve the model by incorporating more data at elevated strain ranges to increase its accuracy and reduce overestimation in future studies.

## Figures and Tables

**Table 1:** Shakedown range boundaries.

Limit	PD between 3000 and 5000 Cycles	$\mathrm{Plastic}\;\mathrm{Strain}\;\mathrm{Rate}\;({\dot{\varepsilon }}_{p})$
Range A–Range B	45 με	2.25 × 10^−2^ με/cycle
Range B–Range C	450 με	2.25 × 10^−1^ με/cycle

**Table 2:** Descriptive characteristics of UGMs.

Sample	Classification ^1^	Grain Size Ratio (%)		MDD (kg/m^3^)	OWC (%)
		≥5 mm	≤80 μm		
1	GW	59.0	4.4	2209	4.23
2	SP	46.0	5.3	2160	4.76
3	GP	62.0	2.2	2107	4.20
4	SP	34.8	7.3	2028	5.00
5	GW	51.0	2.9	2197	6.05
6	SP	12.5	4.7	1880	5.35
7	SP-SM	42.1	6.5	2184	5.80
8	GP	67.5	4.9	2245	5.90
9	SM	28.0	24.0	2170	6.90
10	SM	27.0	32.0	1968	8.80
11	SM	16.9	28.9	2066	4.00
12	SM-SC	20.0	23.0	2160	4.80
13	SM	35.1	19.6	2172	5.00
14	SM-SC	17.0	24.8	2084	5.22

^1^ UGMs were classified using the unified soil classification system, ASTM D2487 [36].

**Table 3:** Proposed stress path.

Sequence 1		Sequence 2		Sequence 3		Sequence 4		Sequence 5	
$\mathrm{Confining}\;\mathrm{Stress},\;\sigma_{3}$ (kPa)	Deviator Stress, q (kPa)	$\mathrm{Confining}\;\mathrm{Stress},\;\sigma_{3}$ (kPa)	Deviator Stress, q (kPa)	$\mathrm{Confining}\;\mathrm{Stress},\;\sigma_{3}$ (kPa)	Deviator Stress, q (kPa)	$\mathrm{Confining}\;\mathrm{Stress},\;\sigma_{3}$ (kPa)	Deviator Stress, q (kPa)	$\mathrm{Confining}\;\mathrm{Stress},\;\sigma_{3}$ (kPa)	Deviator Stress, q (kPa)
20	20	50	120	80	220	110	350	140	510
	50		150		260		400		560
	80		180		300		450		610
	110		210		340		500		660

**Table 4:** Shakedown range boundaries based on post-compaction plastic strain.

Limit	Post-Compaction Plastic Strain Rate (${\dot{\varepsilon }}_{p}^{pc}$)
Range A–Range B	0.154 με/cycle
Range B–Range C	1.544 με/cycle

**Table 5:** Conditions and results on materials used for the validation.

Material	Stress Path	α	β	q (kPa)	p (kPa)	${\dot{\varepsilon }}_{p}^{pc}$ (με)	Shakedown State	
							Table 1	Table 4
UGM-1	1	14.4	0.106	6	19	0.007	A	A
	2	14.5	0.157	10	20	0.014	A	A
	3	39.9	0.098	15	22	0.018	A	A
	4	99.1	0.049	19	29	0.016	A	A
	5	100.4	0.073	25	31	0.029	A	A
	6	103.8	0.087	29	33	0.038	A	A
	7	137.5	0.084	39	44	0.048	A	A
	8	153.4	0.096	47	47	0.065	A	A
	9	96.0	0.171	59	51	0.113	A	A
UGM-2	1	29.8	0.089	28	26	0.011	A	A
	2	35.7	0.096	31	27	0.015	A	A
	3	47.1	0.116	35	28	0.028	A	A
	4	129.1	0.035	39	32	0.014	A	A
	5	95.7	0.101	43	34	0.044	A	A
	6	127.8	0.093	48	35	0.052	A	A
	7	222.2	0.065	53	41	0.054	A	A
	8	162.2	0.124	58	43	0.105	A	A
	9	257.2	0.135	68	46	0.193	B	B
UGM-3	1	14.6	0.108	20	27	0.008	A	A
	2	30.7	0.089	40	33	0.012	A	A
	3	44.1	0.095	60	40	0.019	A	A
	4	38.3	0.162	80	47	0.040	A	A
	5	27.1	0.269	100	53	0.089	A	A
	6	28.2	0.350	120	60	0.194	B	B
	7	74.0	0.295	180	105	0.311	B	B
	8	90.4	0.353	210	115	0.639	B	B
	9	339.0	0.177	200	137	0.429	B	B
	10	416.3	0.180	240	150	0.545	B	B
	11	62.3	0.415	280	163	0.746	B	B
	12	330.2	0.222	300	200	0.684	B	B
	13	48.9	0.447	350	217	0.766	B	B
	14	308.6	0.262	400	283	0.950	B	B

## Data Availability

The data presented in this study are available upon request to the corresponding authors due to confidentiality agreements linked to the research.
